# A deep learning-enabled portable imaging flow cytometer for cost-effective, high-throughput, and label-free analysis of natural water samples

**DOI:** 10.1038/s41377-018-0067-0

**Published:** 2018-09-19

**Authors:** Zoltán Gӧrӧcs, Miu Tamamitsu, Vittorio Bianco, Patrick Wolf, Shounak Roy, Koyoshi Shindo, Kyrollos Yanny, Yichen Wu, Hatice Ceylan Koydemir, Yair Rivenson, Aydogan Ozcan

**Affiliations:** 1 Electrical and Computer Engineering Department, University of California, Los Angeles, CA 90095 USA; 2 Bioengineering Department, University of California, Los Angeles, CA 90095 USA; 3 California NanoSystems Institute (CNSI), University of California, Los Angeles, CA 90095 USA

## Abstract

We report a deep learning-enabled field-portable and cost-effective imaging flow cytometer that automatically captures phase-contrast color images of the contents of a continuously flowing water sample at a throughput of 100 mL/h. The device is based on partially coherent lens-free holographic microscopy and acquires the diffraction patterns of flowing micro-objects inside a microfluidic channel. These holographic diffraction patterns are reconstructed in real time using a deep learning-based phase-recovery and image-reconstruction method to produce a color image of each micro-object without the use of external labeling. Motion blur is eliminated by simultaneously illuminating the sample with red, green, and blue light-emitting diodes that are pulsed. Operated by a laptop computer, this portable device measures 15.5 cm × 15 cm × 12.5 cm, weighs 1 kg, and compared to standard imaging flow cytometers, it provides extreme reductions of cost, size and weight while also providing a high volumetric throughput over a large object size range. We demonstrated the capabilities of this device by measuring ocean samples at the Los Angeles coastline and obtaining images of its micro- and nanoplankton composition. Furthermore, we measured the concentration of a potentially toxic alga (*Pseudo-nitzschia*) in six public beaches in Los Angeles and achieved good agreement with measurements conducted by the California Department of Public Health. The cost-effectiveness, compactness, and simplicity of this computational platform might lead to the creation of a network of imaging flow cytometers for large-scale and continuous monitoring of the ocean microbiome, including its plankton composition.

## Bio-analysis: Rapidly spotting toxicity in a drop of the ocean

A portable device that combines holographic imaging with artificial intelligence can rapidly detect potentially harmful algae in ocean water. Aydogan Ozcan, Zoltan Gorocs and colleagues from the University of California Los Angeles in the United States developed an inexpensive flow cytometer that pumps water samples containing tiny marine organisms, past an LED chip pulsing red, blue, and green light simultaneously. Deep learning algorithms trained to recognize background signals automatically analyze the holographic interference patterns created by the marine organisms and rapidly generate color images with microscale resolution. Sample throughput is boosted 10-fold over conventional imaging flow cytometry by avoiding the use of lenses. Using a lightweight and inexpensive prototype, the team monitored plankton levels at six public beaches and detected a likely toxic organism, the algae Pseudo-nitzschia, at levels matching those from public health laboratories.

## Introduction

Plankton form the base of the oceanic food chain, and thus they are important components of the whole marine ecosystem. Phytoplankton are responsible for approximately half of the global photoautotrophic primary production^1,2^. High-resolution mapping of the composition of phytoplankton over extended periods is very important and but rather challenging because the concentration and composition of species rapidly change as a function of space and time^3^. Furthermore, the factors governing these changes are not fully understood^4^, and phytoplankton population dynamics are chaotic^5^. The changes in the seasonal bloom cycle can also have major environmental^6^ and economic effects^7^. The vast majority of phytoplankton species are not harmful, but some species produce neurotoxins that can enter the food chain, accumulate, and poison fish, mammals, and ultimately humans. Notable examples include *Karenia brevis*, which produces brevetoxin and causes neurotoxic shellfish poisoning^8^; *Alexandrium fundyense*, which generates saxitoxin and causes paralytic shellfish poisoning; *Dinophysis acuminata*, which produces okadaic acid and results in diarrhetic shellfish poisoning^9^; and *Pseudo-nitzschia*, which produces domoic acid and is responsible for amnesiac shellfish poisoning, potentially even leading to death^10,11^. Currently, monitoring of the concentrations of these species in coastal regions, including in California (USA), is usually performed by manual sample collection from coastal waters using plankton nets, followed by transportation of the sample to a central laboratory for light microscopy-based analysis^12^, which is very tedious, slow, and expensive and requires several manual steps performed by professionals.

As an alternative to light microscopy-based analysis, flow cytometry has been used to analyze phytoplankton samples for more than 35 years^13^. The technique relies on using a sheath flow to confine the plankton sample to the focal point of an illuminating laser beam and measuring the forward and side scattering intensities of each individual object/particle inside the sample volume. To aid classification, flow cytometry is usually coupled with a fluorescence readout to detect the autofluorescence of chlorophyll, phycocyanin, and phycoerythrin found in algae and cyanobacteria. Several field-portable devices based on flow cytometry have been successfully used for analyzing nano- and pico-phytoplankton distributions in natural water samples^14–16^. However, taxonomic identification based solely on scattering and fluorescence data is usually not feasible in flow cytometry, and thus these devices are coupled with additional microscopic image analysis^17^ or need to be enhanced with some form of imaging^18,19^. Consequently, *imaging flow cytometry* has become a widely used technique^20^ in which a microscope objective is used to image the sample (e.g., algae) within a fluidic flow. The image capture is triggered by a fluorescence detector, and thus objects with detectable autofluorescence are imaged. Some of the widely utilized and commercially available imaging flow cytometers include the Flowcam^21^ (Fluid Imaging Technologies), Imaging Flowcytobot^22^ (McLane Research Laboratories), and CytoSense^23^ (Cytobouy b.v.). Although these systems are able to perform imaging of the plankton in a flow, they still have some important limitations. The use of a microscope objective lens provides a strong trade-off between the image resolution and volumetric throughput of these systems; therefore, to obtain high-quality images, the measured sample volume is limited to a few milliliters per hour (e.g., 3–15 mL/h). Using lower-magnification objective lenses can scale up this low throughput by approximately tenfold at the expense of image quality. In addition, the shallow depth-of-field of the microscope objective necessitates hydrodynamic focusing of the liquid sample into a few-µm-thick layer using a stable sheath flow. This also restricts the size of the objects that can be imaged (e.g., to <150 µm) as well as the flow velocity and throughput of the system, thus requiring the use of additional expensive techniques such as acoustic focusing^22^. As a result of these factors, currently existing imaging flow cytometers used in environmental microbiology are fairly bulky (weighing, e.g., 9–30 kg) and costly (>$40,000–$100,000), limiting their widespread use.

Holographic imaging of plankton samples provides a label-free alternative to these existing fluorescence-based approaches; in fact, its use in environmental microbiology started more than 40 years ago using photographic films^24^ and subsequently continued via digital cameras and reconstruction techniques^25^. Holography provides a volumetric imaging technique that uses coherent or partially coherent light to record the interference intensity pattern of an object^26^. This hologram can subsequently be reconstructed to digitally bring the object into focus. The hologram contains information on the complex refractive index distribution of the object, and consequently, not only the absorption but also the phase distribution of the sample can be retrieved. There are several implementations of digital holography for imaging a fluidic flow. We can classify these digital holographic microscopy systems in terms of the presence of an external reference wave (in-line^27^ or off-axis^28^), magnification of the imaged volume, and utilization of a lens^29^ or spherical wavefront^30^ for illumination. Off-axis systems can directly retrieve the phase information from the captured hologram; however, their space-bandwidth product and image quality are generally worse than those of in-line systems^26^. Commercially available holographic imaging flow cytometer systems also exist, such as the LISST-Holo2. This platform is a monochrome system (i.e., does not provide color information) and offers relatively poor image quality compared to traditional imaging flow cytometers. The throughput and spatial resolution are coupled in this device, and therefore it can achieve high-throughput volumetric imaging at the cost of limited resolution (~25 µm), which makes it useful for detecting and identifying only larger organisms. Higher-resolution and better image quality systems using microscope objectives in the optical path have also been described in the literature^28,29^. However, the use of microscope objective lenses not only makes these systems more expensive but also limits the achievable field-of-view (FOV) and depth-of-field, thereby drastically reducing the throughput of the system to, e.g., ~0.8 mL/h^31^.

**Fig. 1:**
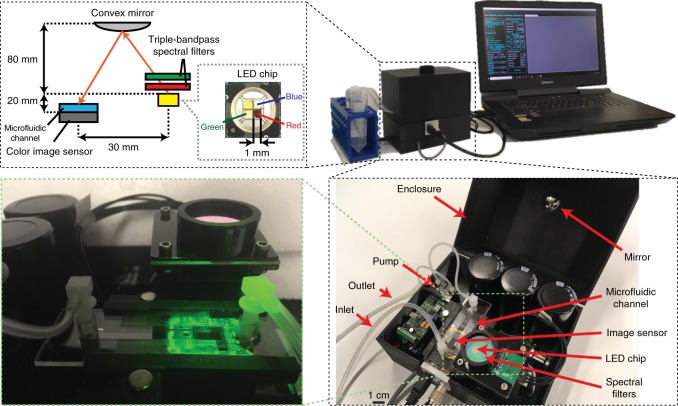
Photos and schematic of the imaging flow cytometer device. The water sample is constantly pumped through the microfluidic channel at a rate of 100 mL/h during imaging. The illumination is emitted *simultaneously* from red, green, and blue LEDs in 120-µs pulses and triggered by the camera. Two triple-bandpass filters are positioned above the LEDs, and the angle of incidence of the light on the filters is adjusted to create a <12 nm bandpass in each wavelength to achieve adequate temporal coherence. The light is reflected from a convex mirror before reaching the sample to increase its spatial coherence while allowing a compact and lightweight optical setup

To provide a powerful and yet mobile and inexpensive tool for environmental microbiology-related research, here we introduce an in-line holographic imaging flow cytometer that is able to automatically detect and provide in real time color images of label-free objects inside a continuously flowing water sample at a throughput of ~100 mL/h. This high-throughput imaging flow cytometer weighs 1 kg with a size of 15.5 cm × 15 cm × 12.5 cm (see Fig. 1) and is based on a deep learning-enabled phase-recovery and holographic-reconstruction framework running on a laptop that also controls the device. Compared with other imaging flow cytometers, the presented device is significantly more compact, lighter weight, and extremely cost-effective, with parts costing less than $2500, only a fraction of the cost of existing imaging flow cytometers. This device continuously examines the liquid pumped through a 0.8-mm-thick microfluidic chip without any fluorescence triggering or hydrodynamic focusing of the sample, which also makes it robust and very simple to operate, with a very large dynamic range in terms of the object size from microns to several hundreds of microns. We demonstrated the capabilities of our field-portable holographic imaging flow cytometer by imaging the micro- and nanoplankton composition of ocean samples along the Los Angeles coastline. We also measured the concentration of the potentially harmful algae *Pseudo-nitzschia*, achieving good agreement with independent measurements conducted by the California Department of Public Health (CDPH). These field results provide a proof-of-principle debut of our compact, inexpensive and high-throughput imaging flow cytometer system, which might form the basis of a network of imaging cytometers that can be deployed for large-scale, continuous monitoring and quantification of the microscopic composition of natural water samples.

## Results

**Fig. 2:**
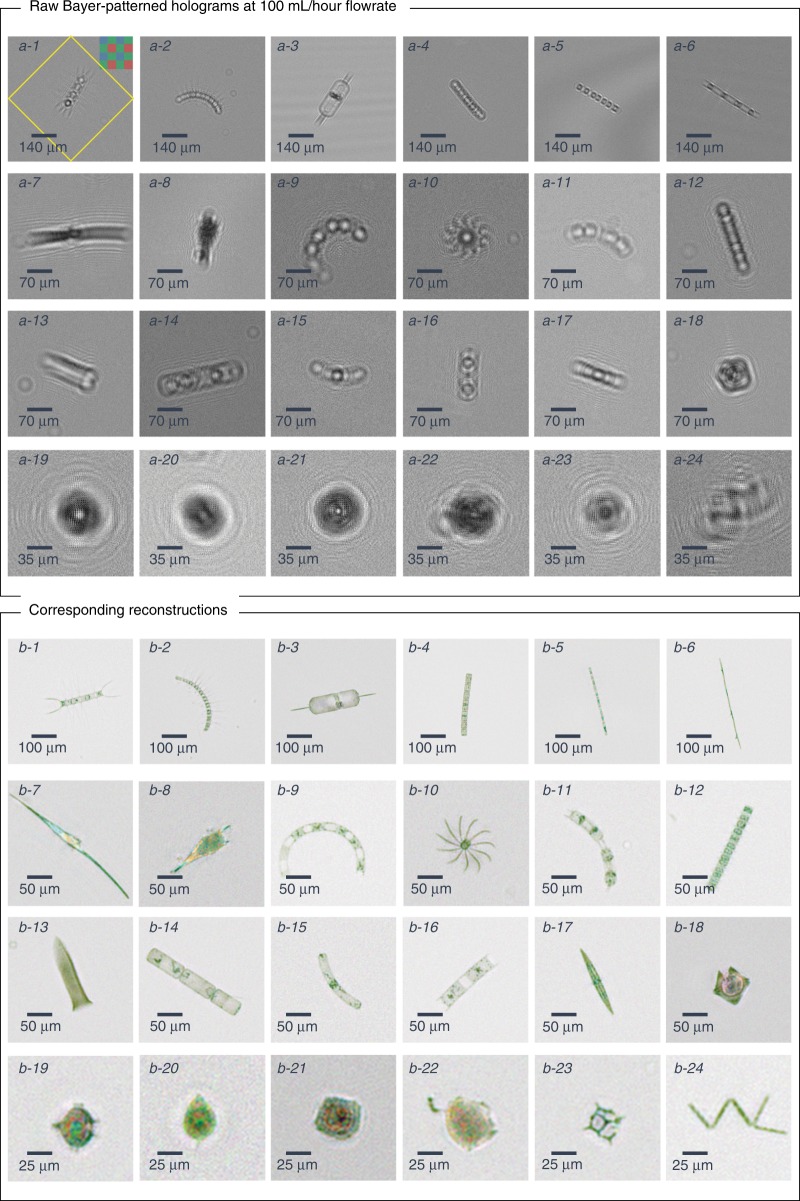
The image quality of the flow cytometer allows the identification of plankton. Examples of various ocean planktons detected by our imaging flow cytometer at the Los Angeles coastline, represented by their **a** raw holograms and **b** phase-contrast reconstructions following phase recovery. The organisms were identified as (1) *Chaetoceros lorenzianus*, (2) *Chaetoceros debilis*, (3) *Ditylum brightwellii*, (4) *Lauderia*, (5) *Leptocylindrus*, (6) *Pseudo-nitzschia*, (7) *Ceratium fusus*, (8) *Ceratium furca*, (9) *Eucampia cornuta*, (10) *Bacteriastrum*, (11) *Hemiaulus*, (12) *Skeletonema*, (13) *Ciliate*, (14) *Cerataulina*, (15) *Guinardia striata*, (16) *Lithodesmium*, (17) *Pleurosigma*, (18) *Protoperidinium claudicans*, (19) *Protoperidinium steinii*, (20) *Prorocentrum micans*, (21) *Lingulodinium polyedra*, (22) *Dinophysis*, (23) *Dictyocha fibula* (silica skeleton), and (24) *Thalassionema*. The yellow rectangle in **a**-1 represents the segmented and 45° rotated area corresponding to the reconstructed images

**Fig. 3:**
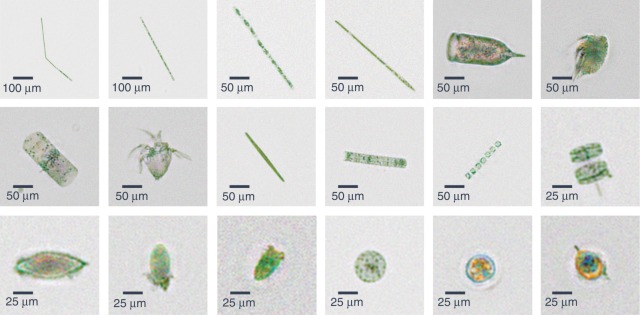
Reconstructed images of various phytoplankton and zooplankton. Phase-contrast color images depicting the plankton found near the Los Angeles coastline and imaged by our flow cytometer at a flowrate of 100 mL/h

**Fig. 4:**
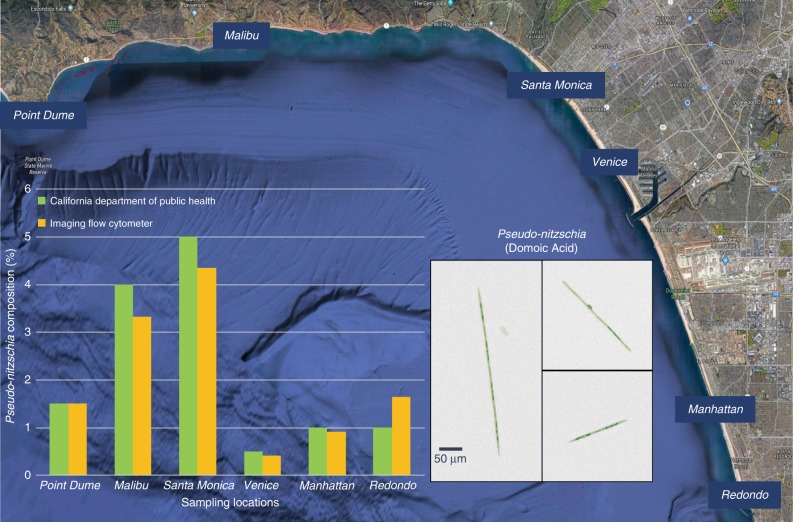
Prevalence of *Pseudo-nitzschia* in the ocean along the Los Angeles coastline on January 31, 2018. Samples were collected according to California Department of Public Health (CDPH) protocols. A portion of each sample was analyzed by the imaging flow cytometer system, and the remainder was sent to CDPH for subsequent analysis, which showed good agreement with our measurements. The inset shows phase-contrast reconstruction examples of *Pseudo-nitzschia*, an alga that can produce domoic acid, a dangerous neurotoxin that causes amnesic shellfish poisoning

We tested our imaging flow cytometer with samples obtained from the ocean along the Los Angeles coastline. The samples were imaged at a flow rate of 100 mL/h, and the raw full FOV image information was saved on the controlling laptop. Plankton holograms were segmented automatically and reconstructed by the device using a deep convolutional network, and phase-contrast color images of plankton were calculated and saved to the local laptop controlling the imaging flow cytometer through a custom-designed graphical user interface (GUI). Figure 2 highlights the performance of this automated deep learning-enabled reconstruction process and the image quality achieved by the device, showcasing several plankton species with both their initial segmented raw images (holograms) and the final-phase contrast color images. We were able to identify most of the plankton types detected by our device based on the reconstructed images, as detailed in the captions of Fig. 2. An additional selection of unidentified plankton imaged in the same ocean samples is also shown in Fig. 3. Some part of the water sample for each measurement was also sent to CDPH for comparative microscopic analysis by their experts, and the qualitative composition of different species found in each water sample was in good agreement with our measurements. Furthermore, to perform a quantitative comparison against the routine analysis performed by CDPH, we selected the potentially toxic *Pseudo-nitzschia* alga and evaluated its relative abundance at six different measurement locations (i.e., public beaches) along the Los Angeles coastline. Our imaging flow cytometer results, summarized in Fig. 4, also showed good agreement with the analysis performed by CDPH. CDPH analyzes the relative abundance of species based on microscopic scanning of a slide containing the settled objects from the water sample of interest, whereas our analysis is based on imaging of the liquid sample itself during its flow. Differences in sample preparation, imaging, and data-processing techniques might cause some systematic differences between the two *Pseudo-nitzschia* composition metrics reported in Fig. 4. However, both methods are self-consistent, and therefore the relative differences that are observed in *Pseudo-nitzschia* composition among different beaches are comparable, illustrating good agreement between our results and the analysis performed by CDPH.

**Fig. 5:**
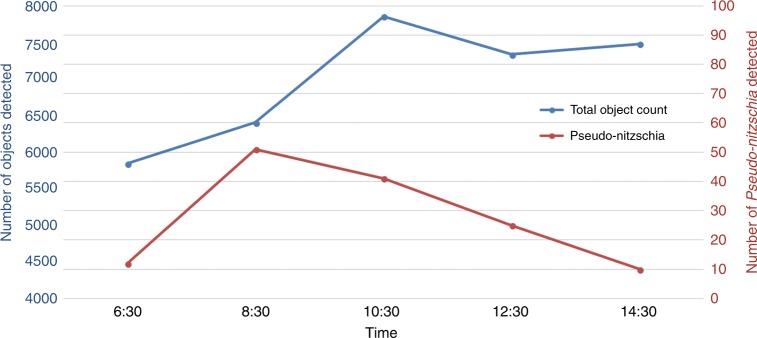
Field test results from a series of measurements at Redondo Beach on April 17, 2018. We sampled the top 1.5 m of the ocean every 2 h and measured on-site the variation in the plankton concentration over time. The measurements started after sunrise (6:21 am), and each sample was imaged on-site using the flow cytometer. The results showed an increase in the total particle count during the day, whereas the number of *Pseudo-nitzschia* showed a peak during the morning hours

We also demonstrated the field portability and on-site operation of our imaging flow cytometer by performing experiments at the Redondo Beach pier over a duration of 8 h. The flow cytometer itself was powered by a 5-V battery pack and could run for several hours. We utilized a 500-Wh 19-V external battery pack to power the laptop for the duration of our field experiments (from 6:30 am until 2:30 pm). In these field experiments, we measured the time evolution of the total plankton concentration in the ocean during the morning hours and found that the amount of microplankton in the top 1.5 m of the water increased during the day, possibly due to vertical migration^32,33^ (see Fig. 5). We also manually counted the number of *Pseudo-nitzschia* found in these samples and observed a peak in the morning (at ~8:30 am) and a steady decline thereafter (Fig. 5); in general, these trends are rather complicated to predict since they are influenced by various factors, such as the composition of the local microbiome, tides and upwelling/downwelling patterns^34,35^. These results demonstrate the capability of our portable imaging flow cytometer to periodically measure and track the plankton composition and concentration of water samples on site for several hours without the need for connection to a power grid.

## Discussion

**Fig. 6:**
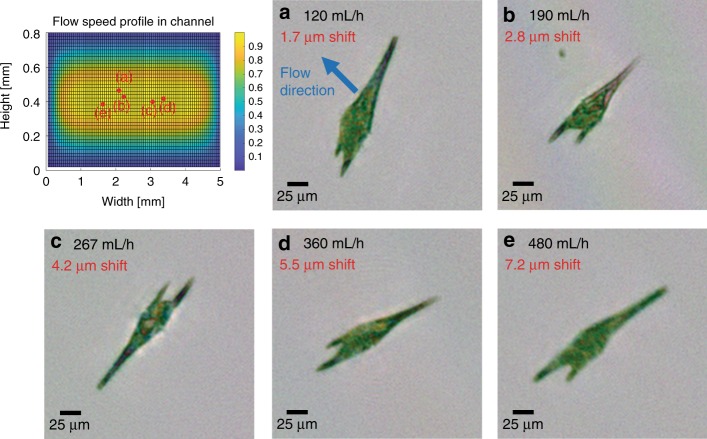
Effect of increasing the liquid flow speed in the system on the image quality. The relative flow speed profile inside the rectangular channel cross-section is depicted in the top left (see the Methods section). The measurements were made on an ocean sample containing a high concentration of *Ceratium furca*, which was therefore used as the model organism for this test. The sample was tested at various flow speeds above 100 mL/h with a constant 120-µs illumination pulse length. We selected the objects located inside the channel near the *maximum*-flow velocity regions, and their locations are depicted as red dots. **a**–**e** Reconstructed intensities corresponding to different flow rates are shown. The flow rate (black) and the theoretically calculated displacement during the illumination pulse (red) are also shown

The throughput of an imaging flow cytometer is determined by several factors, but most importantly it is governed by the required image quality. We designed our portable imaging flow cytometer to achieve the highest resolution allowed by the pixel size of the image sensor, which resulted in a tight photon budget owing to the loss of illumination intensity for achieving sufficient spatial and temporal coherence over the sample volume and the requirement for pulsed illumination to eliminate motion blur. Because of the fast flow speed of the objects within the sample channel, pixel super-resolution^36,37^ approaches could not be used to improve the resolution of the reconstructed images to the subpixel level. We conducted our experiments at 100 mL/h; however, at the cost of some motion blur, this throughput could be quadrupled without any modification to the device. It could be increased even more by using a thicker (e.g., >1 mm) microfluidic channel. To demonstrate this, we imaged an ocean sample with increased throughputs of up to 480 mL/h (see Fig. 6). The obtained reconstructions show that the imaged alga (*Ceratium furca*) still remains easily recognizable despite the increased flow speed.

In addition to the physical volumetric throughput, the processing speed of the controlling laptop can also be a limiting factor, mainly affecting the maximum density of the sample that can be processed in real time. Our device design achieves real-time operation; i.e., the computer processes the information faster than the image sensor provides it to avoid overflowing the memory. Currently, the device can be run in three modes depending on the sample density. First, we can acquire and save the full FOV holograms and perform all reconstruction and phase recovery steps after the measurement, which is a necessary approach for high-concentration samples (e.g., >2000–3000 objects/mL). Even denser samples can also be analyzed by our device by, e.g., diluting them accordingly or by lowering the throughput. Second, we can reconstruct but do not perform phase recovery of the detected objects during measurement. At present, the image-segmentation and reconstruction procedure takes ~320 ms for each full FOV frame, in which seven objects can be reconstructed per image with parallel computing on a GTX 1080 GPU. The major computational operations are (1) segmentation of the full FOV hologram for object detection (~70 ms), (2) holographic autofocusing and reconstruction (~12 ms/object), and (3) transfer of the final amplitude and phase images (8 bit, 1024 × 1024 pixels × 3 color channels) from the device (i.e., GPU) to the host (i.e., central processing unit) and saving of the images on an internal solid-state drive (~10–20 ms per object). Consequently, for reconstruction but not phase recovery of objects, the device can image, in real-time, samples with ~700 objects/mL at a flowrate of 100 mL/h.

The *third* mode of operation of our device involves performing both the image-reconstruction and phase-recovery steps for all flowing objects during the measurement. The deep learning-based phase-recovery step is currently the most intensive part of our algorithm, with a runtime of ~250 ms/object. Thus, if real-time phase recovery is necessary in this third mode of operation, it restricts the sample density to ~100 objects/mL at a flowrate of 100 mL/h. Since the performance of GPUs increases by, on average, 1.5 × per year, these computational performance restrictions will be partially overcome over time.

Furthermore, we have recently shown that is possible to simultaneously focus *all objects* in a hologram using a convolutional neural network^38^ that extends the depth-of-field of holographic reconstruction by >25-fold compared to conventional approaches. This would allow the phase-recovery, autofocusing and image-reconstruction steps to be combined into a single neural network, which would make the computation time for the full FOV *independent* of the density of the particles, thus enabling real-time imaging of highly dense fluidic samples. We tested this approach to reconstruct micro-objects in our 800-µm-thick channel volume and found that it gives good results regardless of the object’s height inside the channel (see Supplementary Figure S1).

Since static objects are removed from our FOV using the digital background-subtraction step, we can tolerate some loss of light transmission due to potential fouling. Since our instrument is based on holography, the amplitude and phase information of a flowing object are dispersed over a larger diffraction pattern at the detector plane, making it robust to potential alterations caused by other small objects in the light path. Furthermore, since the flow chamber of our imaging flow cytometer is disposable, it can be easily replaced if needed; for this purpose, an evaluation of the background image can be used to automatically alert users to the need for replacement.

Although our current prototype is a field-portable imaging flow cytometer, it is not fully waterproof and operates above the water surface. This prototype can operate up to 100 m from the controlling laptop by simply changing the USB3 camera connection to GigE and constructing a long-range microcontroller communication setup similar to an OpenROV^39^ submersible platform. Owing to its low hardware complexity in comparison with other imaging flow cytometer technologies, the component cost of the system is very low (<$2500), and with large volume manufacturing, it could be built for less than $760 (see Supplementary Table S1). This remarkable cost-effectiveness opens up various exciting opportunities for environmental microbiology research and could allow the creation of a network of computational imaging cytometers at an affordable price point for large-scale and continuous monitoring of ocean plankton composition and the ocean microbiome in general.

## Materials and methods

### Optical system

Our imaging flow cytometer uses a color image sensor with a pixel size of 1.4 µm (Basler aca4600-10uc). The housing of the camera is removed, and the circuit is rearranged to allow the sample holder to be placed in direct contact with the protective cover glass of the image sensor (see Fig. 1). Illumination of the holographic microscope is provided by using the red, green, and blue emitters from a light-emitting diode (LED) (Ledengin LZ4-04MDPB). The spatial and temporal coherence of the emitted light from the LEDs is increased to achieve the maximum resolution allowed by the sensor pixel size. The spatial coherence is adjusted by using a convex mirror (Edmund Optics #64-061) to increase the light path. The LED light is also spectrally filtered by two triple-bandpass optical filters (Edmund Optics #87–246, Chroma Inc. 69015m) to increase the temporal coherence of the illumination. The placement of the optical components is designed to tune the bandpass of the spectral filter angle to better match the emission maximum of the LEDs. Increasing the spatial and temporal coherence of the LEDs also decreases the intensity reaching the image sensor. In addition, the short exposure time required to avoid the motion blur when imaging objects in a fast flow makes it necessary for our configuration to utilize a linear sensor gain of 2. The additional noise generated from the gain is sufficiently low as to not interfere with the image-reconstruction process.

### Microfluidic channel and flow design

A microfluidic channel (Ibidi µ-Slide I) with an internal height of 0.8 mm is placed on the top of the image sensor, secured using a three-dimensional (3D)-printed holder, and connected to a peristaltic pump (Instech p625). The size of the active area of the image sensor is slightly smaller than the width of the channel (4.6 mm vs. 5 mm), and the channel is positioned so that the sensor measures the center of the liquid flow. We calculated the flow profile inside the channel (see Fig. 6) by solving the Navier–Stokes equation for noncompressible liquids assuming a nonslip boundary condition. The results show that the image sensor measures ~98% of the total volume passing through the microfluidic channel. The flow profile is a two-dimensional paraboloid, with the maximum flow speed located at the center of the microfluidic channel and measuring approximately 1.66 times higher than the mean velocity of the liquid (see Fig. 6). To acquire sharp, in-focus images of the objects in the continuously flowing liquid, we operate the image sensor in the global reset release mode and illuminated the sample by flash pulses, where the length of an illuminating pulse is adjusted to not allow an object traveling at the maximum speed inside the channel to shift by more than the width of a single sensor pixel. For a flowrate of 100 mL/h, this corresponds to a pulse length of 120 µs.

### Pulsed illumination, power, and control circuit

Because shortening the illumination time also constrains the available photon budget, we maximize the brightness of our LEDs by operating them at currents ranging from 2.2 to 5 A depending on their color. The currents are set for each LED emitter to create similar brightness levels at our image sensor, ensuring that we adequately light the sample at each color, a requirement for obtaining color images. The green LED spectrum is inherently wider than the red and blue counterparts, and thus the spectral filters will reduce its intensity the most. Therefore, we operate the green LED at the experimentally determined maximum possible current of 5 A. The red and blue LEDs require a current of ~2.2 A to match the intensity of the green LED on the image sensor to correct the white balance. We designed a circuit to control the necessary components of the device. The circuit is powered by either a 5-V wall-mounted power supply or a cellphone charger battery pack. The circuit fulfills four major roles: providing power to the peristaltic pump, charging the capacitors for providing power to the LEDs, synchronizing the LEDs to the camera and creating stable, short, high current pulses, and, finally, providing an interface for remote control by a laptop using an Inter-Integrated-Circuit (i2c) interface for setting various parameters. The peristaltic pump is powered by a high-efficiency step-up DC-DC converter at 16 V (TPS61086, Texas instruments), and its speed is controlled by a potentiometer via i2c components (TPL0401B, Texas Instruments). The charge for the high-current pulses is stored in three 0.1-F capacitors, which are charged using a capacitor charger controller (LT3750, Linear Technologies) to 12 V. The capacitor charge is initiated by the image sensor flash window trigger signal, which is active during the frame capture, and its length can be controlled by the camera software driver. The charger controller acquires an “on” state and keeps charging the capacitors until the preset voltage level of 12 V is reached. During the short illumination pulses, the voltage on the capacitors decreases only slightly, and they are immediately recharged as each frame capture resets the charge cycle, thereby allowing continuous operation. The LEDs are synchronized and their constant-current operation is ensured by a triple-output LED driver controller (LT3797, Linear Technologies). The controller uses the same flash window signal from the image sensor to turn on the LEDs for the exposure duration set by the software. The current of each LED is controlled between 0 and 12.5 A using digital i2c potentiometers (TPL0401A, Texas Instruments) and is kept constant for the subsequent pulses by the circuit, thus maintaining the same illumination intensity for each holographic frame. During startup, it takes ~3–4 frames for the circuit to stabilize at a constant light level. To avoid having multiple devices with the same address on the i2c line, we included an address translator (LTC4317, Linear Technologies) to interface with the potentiometers controlling the red and blue LEDs. To control the circuit, the laptop communicates with an Arduino microcontroller (TinyDuino from Tinycircuits), which is used as an interface for i2c communications only. During the initial startup, the circuit consumes ~1 A for the first ~8 s until the capacitors are fully charged. During its operation, the circuit consumes, on average, ~370 mA with a pump speed setting of 100 mL/h and a frame rate of 3 frames per second. This yields an average power consumption of less than 2 W. In addition, the image sensor’s typical power consumption is ~2.8 W according to the manufacturer’s data.

### Object detection and deep learning-based hologram reconstruction

**Fig. 7:**
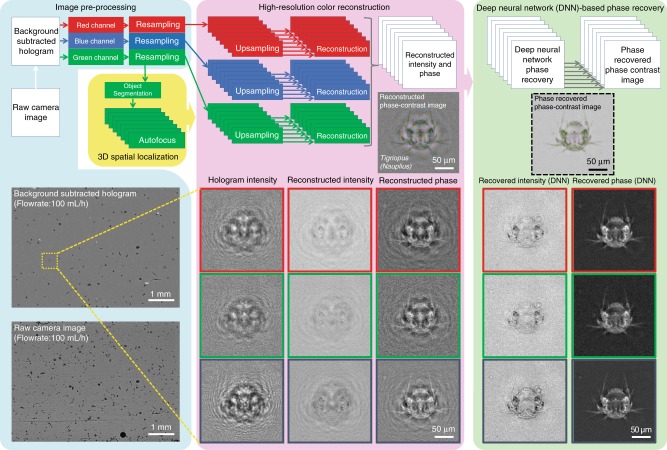
The algorithm used for object segmentation and deep learning-based hologram reconstruction in our field-portable imaging flow cytometer is illustrated. The phase-recovered intensity and phase images in red, green, and blue channels are fused to generate a final phase-contrast image per object (shown within the dashed black frame on the right)

For automatic detection and holographic reconstruction of the target objects found in the continuously flowing water sample (see Fig. 7), the static objects found in the raw full FOV image (e.g., dust particles in the flow channel) need to be eliminated first. This is achieved by calculating a time-averaged image of the preceding ~20 images containing only the static objects and subtracting it from the present raw hologram. To ensure appropriate reconstruction quality, the mean of this subtracted image is added back uniformly to the current frame. This yields a background-subtracted full FOV image in which only the holograms of the objects newly introduced by the flow are present. These objects are automatically detected and segmented from the full FOV for individual processing (see Supplementary Figure S2). The full FOV background-subtracted hologram is first Gaussian-filtered and converted into a binary image by hard-thresholding with its statistical values (mean + 1.5 × standard deviation), which isolates the peaks of the holographic signatures created by the objects included in the FOV. The binary contours with an area of a few pixels are removed to reduce misdetection events due to sensor noise. A closing operation is performed in the generated binary image to create a continuous patch for each object. The resulting binary contours represent the shapes and locations of the objects appearing in the FOV, and their morphological information is used to filter each contour by certain desired criteria (e.g., major axis). The center coordinate of the filtered contour is used to segment its corresponding hologram. We should emphasize that not only is it feasible to extract all objects in the FOV but it is also possible to prioritize the segmentation of the objects of interest for a specific goal by our approach. Thus, we can better utilize the computational resources of the laptop and maintain real-time processing for denser samples. After segmentation, the Bayer-patterned holograms are separated into three mono-color (i.e., red, green, and blue) holograms corresponding to the illumination wavelengths. To fully utilize the spatial resolution of the optical system, the orientation of the Bayer-patterned green pixels is rotated by 45° to regularize their sampling grid^40^. Concurrently, the red and blue mono-color holograms are upsampled by a factor of two, and a 45° rotation is applied to these upsampled holograms. These processes are jointly called “Resampling” in Fig. 7. Holographic autofocusing using the Tamura coefficient of the complex gradient^41,42^ is performed for each segmented object using only a single mono-color hologram to accurately estimate the distance of the respective object from the image sensor plane. At this point, we have 3D localized each object within the flow (per FOV). The coordinates of each detected object are then used in conjunction with the estimated flow profile from our calculations, and the location of each object is predicted at the next frame. If an object is found at the predicted coordinates, it is flagged to be removed from the total count and processing workflow to avoid reconstructing and counting the same object multiple times. At this point, the image preprocessing step (shown in cyan in Fig. 7) is complete.

The next step is the high-resolution color reconstruction (shown in pink in Fig. 7). We maximize the resolution of the reconstruction by further upsampling the holograms by a factor of four. Each color channel is then propagated to the obtained reconstruction distance by an angular spectrum-based wave-propagation algorithm^26^ and thus brought into focus. The slight incidence angle difference between the red, green, and blue emitters is corrected by modifying the propagation kernel accordingly^43^. To evaluate the resolution of the imaging flow cytometer system for the objects located inside our microfluidic channel, we replaced the flow channel with a 1951 Air Force test chart (see Supplementary Figure S3). Due to the partially coherent nature of our illumination, the resolution depends on the object–sensor distance; thus, we measured it by placing the test chart at various heights above the sensor. The width of the smallest resolved line varied between 1.55 µm and 1.95 µm depending on the height of the object, with 1.55 µm corresponding to the smallest resolvable feature for most flowing objects imaged by the imaging flow cytometer during its regular operation.

These raw reconstructions, however, are contaminated by self-interference and twin-image noise, which are characteristic of in-line digital holographic imaging systems due to the loss of the phase information of the hologram at the sensor plane. To achieve accurate image reconstruction without these artifacts, a deep learning-based digital holographic phase recovery method^38,44^ was employed using a convolutional neural network (see Fig. 7 and Supplementary Figure S4) pretrained with various phase-recovered reconstructions of water-borne micro-objects captured with our imaging flow cytometer. This method enables automated and accurate acquisition of the spectral morphology of an object without sacrificing the high-throughput operation of the holographic imaging cytometer, which otherwise would be very challenging as other existing phase-recovery methods require static repetitive measurements^43,45–48^ and/or time-consuming iterative calculations^43,45–50^, which would not work for flowing objects. For the visualization of transparent objects, such as plankton, we computed the color phase-contrast image based on the complex-valued reconstructions of the red, green, and blue channels, which assist in accurately resolving the fine features and internal structures of various water-borne microorganisms with high color contrast (see, e.g., Figs. 2, 3, and 7). The phase-contrast image was synthesized by (1) estimating the background field from the mean amplitude and phase of the refocused complex field, (2) calculating the object field by subtracting the background field from the refocused field, (3) shifting the phase of the background field by *π*/2, (4) adding the phase-shifted background field to the object field, and (5) taking the magnitude of the recalculated total field.

### Graphical user interface

We developed a GUI to operate the device. Through this GUI, all relevant measurement parameters can be specified, such as the liquid flow speed, the driving currents, the incidence angles for the red, green, and blue LEDs, the flash pulse duration, and the camera sensor gain. The GUI gives a real-time, full FOV reconstructed image at the center of the channel, thus allowing visual inspection during flow with and without background subtraction, and displays the total number of detected objects in the current frame. The GUI is also capable of visualizing up to 12 segmented, autofocused, and reconstructed objects in real time. The user can specify whether to digitally save any combination of the raw, background-subtracted holograms or reconstructed images. The GUI can also be run in demo mode to analyze previously captured image datasets without the presence of the imaging flow cytometer.

### Sample preparation and analysis

We followed the sampling protocol recommended by CDPH (USA) to obtain our ocean samples. We used a plankton net with a diameter of 25 cm and a mesh size of 20 µm and performed vertical tows with a total length of 15 m (5 × 3 m) from the end of the pier at each sampling location where a pier was present (Malibu, Santa Monica, Venice, Manhattan, and Redondo; California, USA). There was no pier at Point Dume; thus, we performed a horizontal tow from the shoreline. The plankton net condensed the micro- and nanoplankton found in the ocean into a sample volume of ~250 mL; i.e., in our case, a condensation ratio of ~3000×. We extracted 1 mL of the condensed sample, rediluted it with 50 mL of filtered ocean water, and imaged its contents using our imaging flow cytometer. The remaining samples were sent to CDPH for subsequent analysis (used for comparison purposes). During our field tests, we used the same plankton net but only performed one vertical tow from a depth of 1.5 m at each measurement. A 1-mL aliquot of the obtained sample was rediluted with 20 mL of filtered ocean water. To conserve the battery power of the controlling laptop, ~12 mL of this sample was imaged on-site. The imaging flow cytometer automatically detected and saved the reconstructed images of all detected plankton and provided the user real-time feedback on the total plankton count detected. Specific counting of *Pseudo-nitzschia* was performed manually by scanning through the dataset of the saved images and visually identifying *Pseudo-nitzschia*.

## Electronic supplementary material

Supplementary Information
