# Supplementary material for: A deep learning-enabled portable imaging flow cytometer for cost-effective, high-throughput, and label-free analysis of natural water samples
Source: Light Sci Appl. 2018 Sep 19;7:66. doi: 10.1038/s41377-018-0067-0 (PMC6143550; doi:10.1038/s41377-018-0067-0)
Supplement: Supplementary file 1 — Supplementary Information [file 41377_2018_67_MOESM1_ESM.pdf]

Supplementary information for

A deep learning-enabled portable imaging flow-cytometer for cost-effective, high-throughput and label-free analysis of natural water samples

Zoltán Göröcs[1,2,3,†], Miu Tamamitsu[1,2,3,†], Vittorio Bianco[1], Patrick Wolf[1], Shounak Roy[1], Koyoshi Shindo[1], Kyrollos Yanny[2], Yichen Wu[1,2,3], Hatice Ceylan Koydemir[1,2,3], Yair Rivenson[1,2,3], and Aydogan Ozcan[1,2,3,*]

[1]*Electrical and Computer Engineering Department,* [2]*Bioengineering Department, and* [3]*California NanoSystems Institute (CNSI),*
University of California, Los Angeles, CA, 90095, USA.

**ozcan@ucla.edu*

[†]*These authors contributed equally*

Supplementary Figures

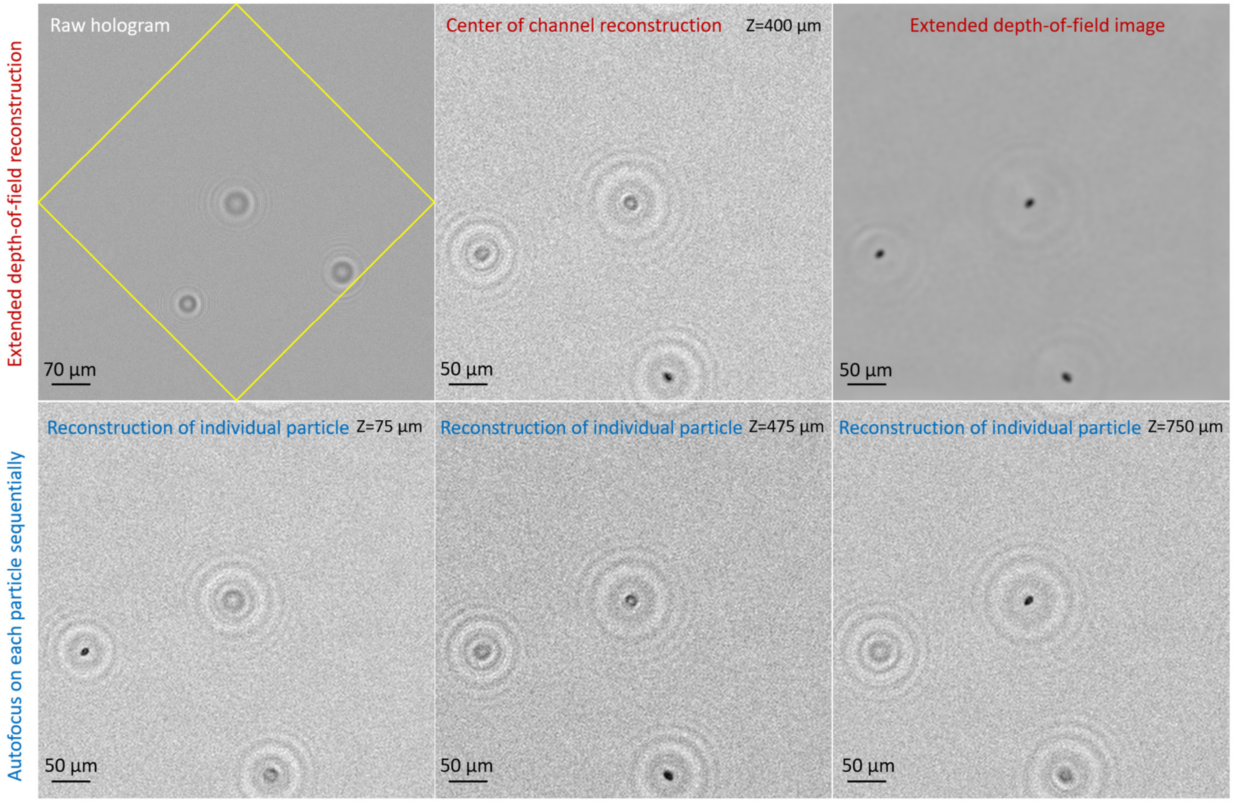

Figure S1: Deep learning-based extended depth-of-field (DOF) reconstruction of flowing *Giardia* cysts. (Top) The raw hologram captured by the image sensor is separated into individual color channels and reconstructed at the height, approximately corresponding to the center of the channel. This initial reconstruction is used as an input for a deep neural network trained to reconstruct holograms *irrespective* of their object heights in a single step, automatically implementing the function of both auto-focusing and phase recovery; thereby generating an extended depth-of-field image of the scene by *simultaneously* reconstructing and bringing into focus all the particles. (Bottom) Individual reconstructions of the same raw hologram using autofocusing on each particle. Particles reconstruct at different heights spanning the height of the flow channel (0–800 µm); this comparison between the top and bottom rows clearly shows that the whole volume can be coalesced into a single plane using a deep neural network based extended DOF reconstruction (top right image), enabling the reconstruction of dense water samples without being bottlenecked with the local computational power that is available.

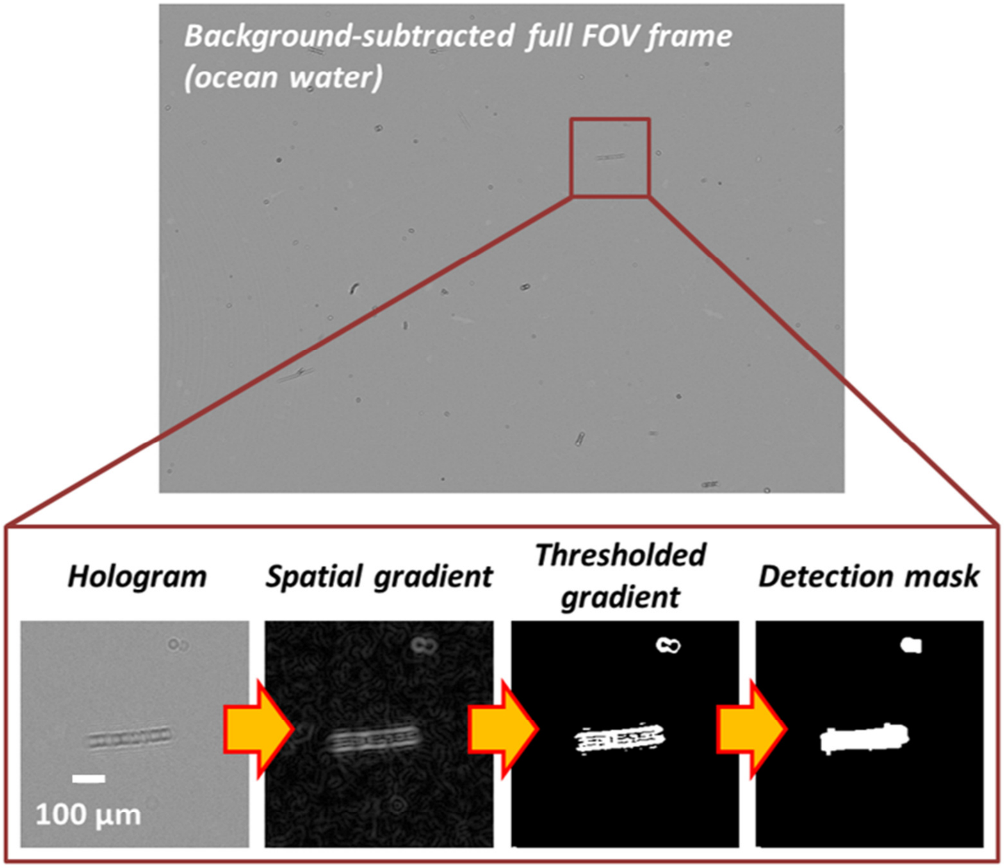

Figure S2: Segmentation algorithm utilized by our imaging flow cytometer for automated plankton detection. The spatial gradient of the full field-of-view background-subtracted hologram is calculated to detect the rapidly oscillating holographic diffraction pattern of the plankton present in the image. The gradient is thresholded to create a binary image, and morphological closing is performed to obtain a single mask signature from each object. The center coordinates of the masks are calculated and used to segment the full field-of-view hologram into sub-holograms containing a single organism/object.

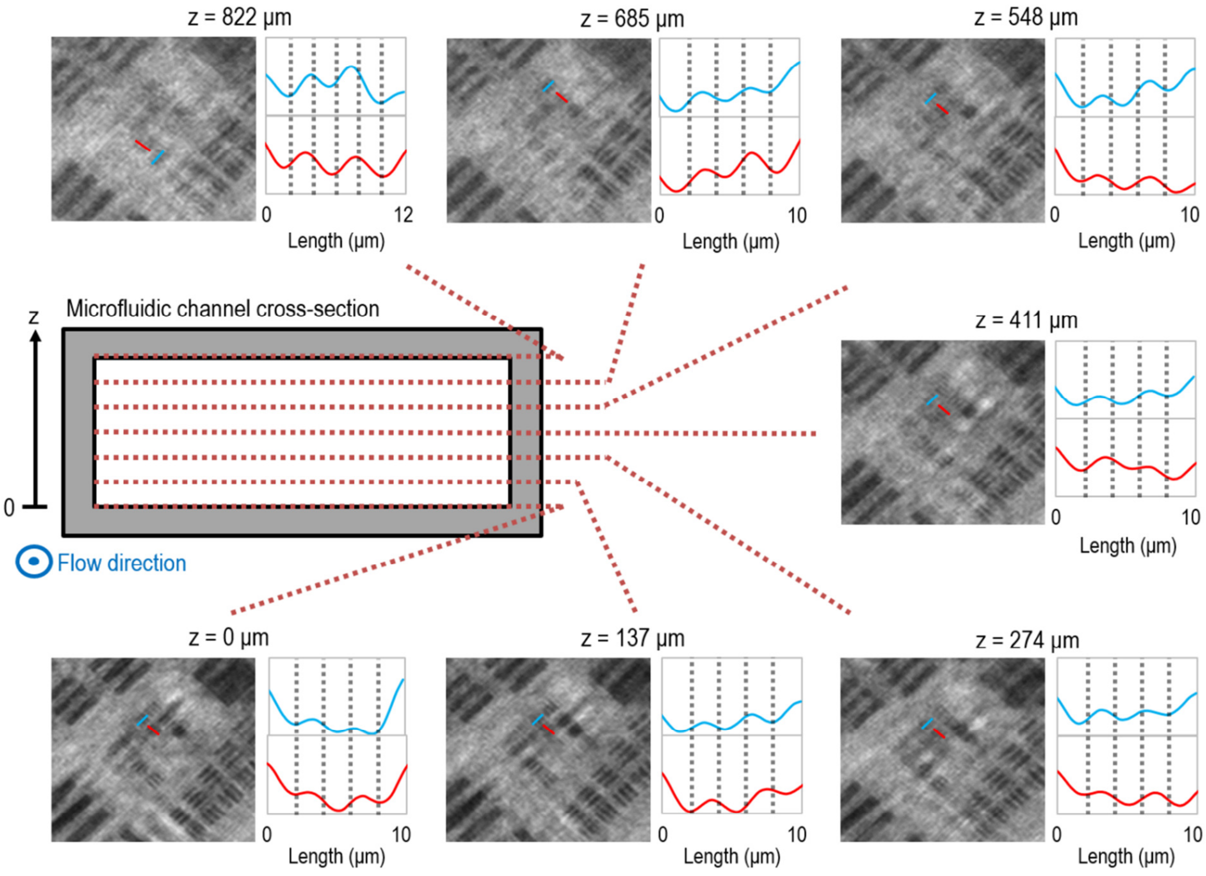

Figure S3: Imaging performance of our color holographic imaging flow cytometer. A 1951 Air Force test chart was placed at seven different distances (z) from the CMOS sensor plane corresponding to the height range of the microfluidic channel (i.e., z=0 corresponds to the bottom of the water sample in the channel). The smallest resolved element on the chart up to ~550 µm height is group 8 element 3, corresponding to a linewidth of 1.55 µm. Above this height, the coherence of the light reduces the achievable resolution steadily with z distance, with the top of the channel resolving a linewidth of 1.95 µm corresponding to group 8 element 1.

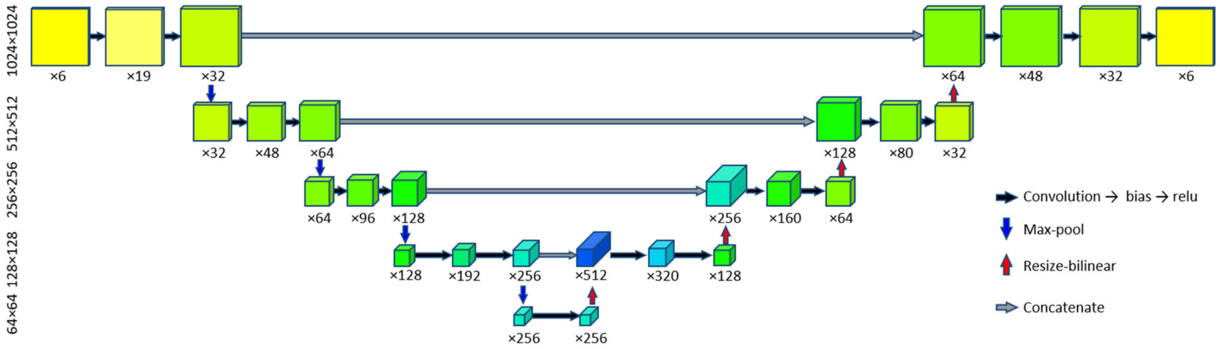

Figure S4: Architecture of the convolutional neural network (CNN) used for holographic image reconstruction. The input matrix is 1024 × 1024 pixels each, for RGB intensity (×3) and RGB phase channels (×3), i.e., altogether forming 6 channels. The network output is the phase-recovered and twin-image eliminated RGB intensity and RGB phase of the flowing object.

Component	Single Unit (USD)	High Volume (USD)
Pump	700	~420
Image Sensor	676	~115
Illumination Circuit	~300	~110
Optical Filters	400+375	<100
Flow Channel	~15	<10
Total	~2466	<755

Table S1: Cost estimate (in USD) for the components of our imaging flow cytometer prototype.
